# MAFCO: A Compression Tool for MAF Files

**DOI:** 10.1371/journal.pone.0116082

**Published:** 2015-03-27

**Authors:** Luís M. O. Matos, António J. R. Neves, Diogo Pratas, Armando J. Pinho

**Affiliations:** Signal Processing Lab, IEETA/DETI, University of Aveiro, 3810–193 Aveiro, Portugal; University of California-Riverside, UNITED STATES

## Abstract

In the last decade, the cost of genomic sequencing has been decreasing so much that researchers all over the world accumulate huge amounts of data for present and future use. These genomic data need to be efficiently stored, because storage cost is not decreasing as fast as the cost of sequencing. In order to overcome this problem, the most popular general-purpose compression tool, gzip, is usually used. However, these tools were not specifically designed to compress this kind of data, and often fall short when the intention is to reduce the data size as much as possible. There are several compression algorithms available, even for genomic data, but very few have been designed to deal with Whole Genome Alignments, containing alignments between entire genomes of several species. In this paper, we present a lossless compression tool, MAFCO, specifically designed to compress MAF (Multiple Alignment Format) files. Compared to gzip, the proposed tool attains a compression gain from 34% to 57%, depending on the data set. When compared to a recent dedicated method, which is not compatible with some data sets, the compression gain of MAFCO is about 9%. Both source-code and binaries for several operating systems are freely available for non-commercial use at: http://bioinformatics.ua.pt/software/mafco.

## Introduction

Certainly motivated by the dramatic drop in price in genomic sequencing, the research community is continuously increasing the volume of sequenced data. Unfortunately, the pace at which storage and communication resources are evolving is not enough to compensate this tendency. Several functional studies have been focusing on annotating the genomes of human and model organisms. One possible approach to facilitate these studies is to use Whole Genome Alignments.

Whole Genome Alignments tend to be very large, occupying several hundreds of gigabytes of disk space and containing millions of alignment blocks. Handling data at this scale presents several challenges in download speed and storage space. The problem of compressing massive amounts of genomic data is addressed in [1, 2] and is currently one of the most intensive research topics in the field of data compression. In the last two decades, several specialized algorithms for compressing DNA sequences and several other forms of genomic data were developed and used by the research community (e.g., [3–15]). Most of these algorithms take into account only the four-letter alphabet, ACGT. However, other letters outside the main alphabet, although usually less frequent, need also to be represented, if the original data are to be recovered. In fact, there is the possibility of having lower case letters, such as acgt, or even other symbols (e.g. n/N). In order to create a fully lossless compression algorithm, all these symbols must be taken into account. Furthermore, it is also necessary to consider additional data, such as headers, quality scores or alignment information.

Recently, we have proposed an algorithm, based on a mixture of finite-context models and arithmetic coding, for compressing only the MSABs (Multiple Sequence Alignment Blocks) of the MAF [16] files [11]. This algorithm was only designed to compress the DNA bases and alignment gaps that are present in the MSABs. All the optional lines and header information were not considered. Furthermore, non-ACGT letters (including lower case) were also not considered. Therefore, the approach described in [11] is lossy in the sense that it does not permit the full recovery of the original file. The MAFCO tool described in this paper is a fully lossless compression method, capable of handling all data found in a MAF file. MAFCO handles all lower case letters, non-ACGT letters, and all the header data and optional lines that were not considered previously.

The remainder of the paper is organized as follow. In Section 1.1, we describe the format of the data sets known as whole genome alignments. In Section 1.2, we describe the existing methods specially designed to compress MAF files. Section 2 contains a description of the algorithm presented in this paper and also the data sets used to evaluate the performance of it. The experimental results for several compression tools are presented in Section 3. Finally, in Section 4, we draw some conclusions.

### 1.1 Whole genome alignments

Computational genome annotation and evolutionary genomics are two molecular biology research areas that use multiple genome alignment data. The alignment of DNA sequences has been used to help locating certain kinds of functional non-coding regions [17] and more recently for finding protein-coding genes [18, 19] and non-coding RNA genes [20]. Moreover, it is possible to observe the similarities and differences between the DNA sequences of humans and other species that share a common ancestral, providing critical data for finding the course of evolution. Furthermore, we can also perform a computational reconstruction of ancestral genome sequences that explains certain characteristics of species [21]. DNA sequences that have evolved from the same ancestral sequence are called homologous. In the case of genes, they are likely to encode similar functions and each function that is experimentally verified in one species can be mapped to a homologous gene in other species.

**Fig 1:**
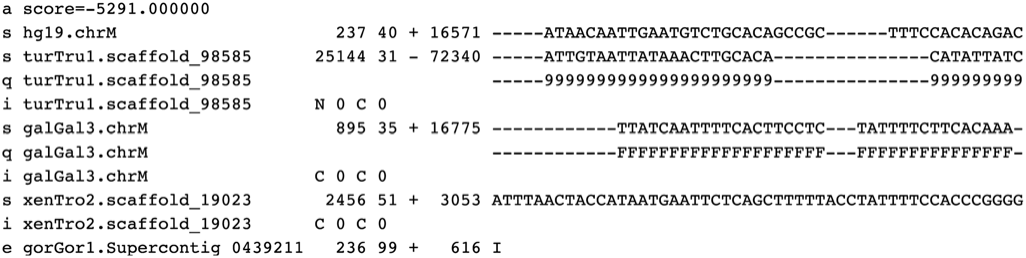
An example of a MSAB with ‘s’, ‘q’, ‘i’, and ‘e’ lines. The ‘s’ lines are the most important lines containing the sequence alignment (DNA bases and gaps ‘-’). The ‘q’, ‘i’, and ‘e’ lines are optional so they can be present or not in a MAF file.

**Fig 2:**
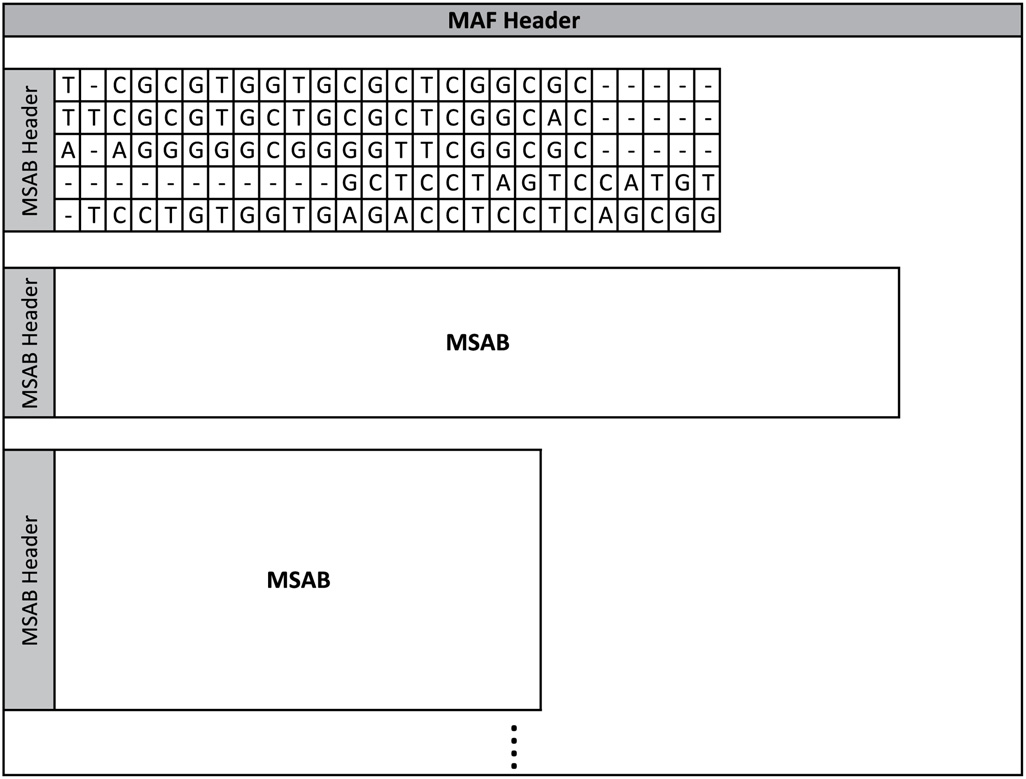
A basic description of a MAF file. Each Multiple Sequence Alignment Block (MSAB) contains the DNA bases for a set of species properly aligned, using an additional gap ‘-’ symbol. The MSABs can also contain additional information that although not mentioned in this figure, it is mentioned latter in this section.

As mention before, this particular voluminous data set in molecular genomics, known as whole genome alignments, has gained considerable importance over the last years. In this context there is one format, called Multiple Alignment Format (MAF) [16], that is used to store a series of multiple alignments of several species and chromosomes. In Fig. 1 we depict an example of a Multiple Sequence Alignment Block (MSAB) with several line types. Each MAF file contains several MSABs, as can be seen in Fig. 2. Those MSABs are derived from the genome rearrangements caused by large scale mutations. We can find alignments of whole genomes in large databases, such as those of UCSC [22] and Ensemble [23]. Each MSAB contains several types of lines as can be seen in Fig. 1. In the following section we describe in more detail the MAF format and the line types that are part of it.

#### 1.1.1 Multiple Alignment Format (MAF)

The multiple alignment format (of MAF format) is used for storing a series of multiple alignments in a format that is easy to parse and relatively easy to read. This format is used to store multiple alignments at the DNA level between entire genomes. A MAF file is composed by several MSABs (Multiple Sequence Alignment Blocks). Each one of those MSABs contain several types of lines. The MSAB always starts with an ‘a’ line that contains the score information. Usually, this line type defines the beginning of a new MSAB. The ‘s’ lines are the most important lines, containing information about the sequence alignment (DNA bases and gaps). The first ‘s’ line of each MSAB is the reference sequence from which the alignment was made. According to [16], the format of a ‘s’ line is:

s [*source*] [*start*] [*size*] [*strand*] [*srcSize*] [*MSA*]

The ‘s’ lines have several fields which are described next:

- [*source*] contains the name of one of the source sequences for the alignment. This field is usually defined as [*specieName.chromossome*] or [*specieName.scaffold*].
- [*start*] the start position of the aligning region in the source sequence.
- [*size*] the size of the aligning region in the source sequence. This number corresponds to the number of non-gaps in the MSAB alignment.
- [*strand*] defines if the alignment is done to the reverse-complement source.
- [*srcSize*] the size of the entire source sequence, not just the parts involved in the alignment.
- [*MSA*] the nucleotides and gaps in the alignment.

The ‘q’, ‘i’, and ‘e’ line types are optional, so there are MAF files that might not have them. The ‘q’ lines contain information regarding the quality of each aligned base for the current source sequence. This quality information is a compressed version of the actual raw quality data, where the quality of each aligned DNA base is represented using a single character that can be a number between 0 and 9 or ‘F’ which represents a finish sequence (see S1 Table for more details). These ‘q’ lines are always associated with the previous ‘s’ lines and therefore they share the same source name. The first ‘s’ line (reference source sequence) of each MSAB does not have ‘q’ lines associated. The ‘q’ lines have the following structure:

q [*source*] [*qualityValues*]

The ‘q’ lines have only two fields:

- [*source*] contains the name of the source sequence. Should be the same source name as the ‘s’ line immediately preceding this line.
- [*qualityValues*] the MAF quality values that correspond to the aligning DNA bases in the previous ‘s’ line. The alignment gap (‘-’) that is present in the previous ‘s’ line is replicated to the ‘q’ line as well. The quality values are computed using
  $$\mathrm{MAFQV}=\min\left(\left\lfloor \frac{\mathrm{AQV}}{5}\right\rfloor ,9\right),$$ (1)
  where MAFQV denotes the MAF quality value that is used in the ‘q’ lines and the AQV represents the actual quality value. S1 Table shows the range of quality values for the ‘q’ lines, as well as the mapping of the raw quality values to the MAF quality values.

The ‘i’ lines contain information about what is happening before and after this MSAB in the aligning source. These informative lines have information about the context of the source sequence lines immediately preceding them. The ‘i’ lines have the following format:

i [*source*] [*lStatus*] [*lCount*] [*rStatus*] [*rCount*]

Similar to the ‘q’ lines, the source name of a ‘i’ line is the same one as of the preceding ‘s’ line. The possible ‘i’ line fields are:

- [*source*] contains the name of the source sequence. Should be the same source name as in the ‘s’ line immediately preceding this line.
- [*lStatus*] left status is represented by a single character that describes the relationship between the sequence in this MSAB and the sequence that appears in the previous MSAB.
- [*lCount*] left count is usually the number of DNA bases in the aligning source between the start of this alignment and the end of the previous one.
- [*rStatus*] right status is represented by a single character that describes the relationship between the sequence in this MSAB and the sequence that appears in the subsequent MSAB.
- [*rCount*] right count is usually the number of DNA bases in the aligning source between the end of this alignment and the start of the next one.

The status characters that can be found in ‘i’ lines can only have one of the following values:

- ‘C’—the sequence before or after is contiguous to this MSAB.
- ‘I’—there are bases between the bases in this MSAB and the one before or after it.
- ‘N’—this is the first sequence from this source.
- ‘n’—this is the first sequence from this source but it is bridged by another alignment from a different chromosome/scaffold.
- ‘M’—there is missing data before or after this block (‘N’ or ‘n’ symbols in the sequence).
- ‘T’—the sequence in this MSAB has been used before in a previous MSAB (likely a tandem duplication).

Finally, the ‘e’ lines contain information about empty parts of the MSAB. The ‘e’ lines indicate if there is not aligning DNA for the specified source, but the current MSAB is bridged somehow by a chain that connects MSABs before and after this MSAB. The ‘e’ line has the following structure:

e [*source*] [*start*] [*size*] [*strand*] [*srcSize*] [*status*]

The fields of the ‘e’ lines are described next. There are some fields that are very similar to the ones of the ‘s’ lines described earlier.

- [*source*] contains the name of one of the source sequences for the alignment.
- [*start*] the start position of the non-aligning region in the source sequence.
- [*size*] the size of the non-aligning region in the source sequence.
- [*strand*] defines if the previous alignment is done to the reverse-complement source.
- [*srcSize*] the size of the entire source sequence, not just the parts involved in the alignment.
- [*status*] a character that specifies the relationship between the non-aligning sequence in this MSAB and the sequence that appears in the previous and subsequent MSABs.

The status characters that can be found in ‘e’ lines can only have one of the following values:

- ‘C’—the sequence before or after is contiguous implying that this region was either deleted in the source or inserted in the reference sequence.
- ‘I’—there are non-aligning bases in the source species between chained MSABs before and after this MSAB.
- ‘N’—there are non-aligning bases in this source and the next MSAB starts in a new chromosome or scaffold that is bridged by a chain between still other MSABs.
- ‘M’—there is missing data before or after this block (‘N’ or ‘n’ symbols in the sequence).
- ‘T’—the empty region of this MSAB has been used before in a previous MSAB (likely a tandem duplication).

### 1.2 Related work

To the best of our knowledge, there is only one specialized tool available for the compression of MAF files [24]. That method is based on well-established statistical evolutionary models and on prediction techniques used for lossless binary image compression. In their proposed compression model, the nucleotides in a MSAB are compressed using the predictions obtained from a nucleotide substitution model, whereas the gaps are encoded independently using techniques from lossless binary image compression. According to the authors, encoding the nucleotides and the gaps separately is justified by the independence of the two underlying mutational processes and should not introduce an inherent loss to the achievable compression rate.

In 2012, Wheeler and Tarasov developed a plugin for BioRuby that offers support to deal with Multiple Alignment Format (MAF) files [25–27]. This plugin provides a set of tools for indexed and sequential access to MAF data, as well as for performing various manipulations on it and writing modified MAF files. In particular, this library provides support for BGZF (Blocked GZip Format) compressed MAF files, which combines gzip compression with blocking for efficient random access. The maf-bgzip tool creates compressed MAF files that consist of concatenated 64 kilobytes blocks, each one as an independent gzip stream. These files can be decompressed entirely with gzip. However, this library enables random access using “virtual offsets” as defined in SAM/BAM for fast access to a certain portion of the MAF file. This tool is not optimized in terms of compression (as it can be seen in the results section). The goal of the tool is to improve the random access of gziped files, sacrificing substantial compression performance.

In 2013, we proposed a compression algorithm for the ‘s’ lines of the MSABs [11], based on a mixture of finite-context models and arithmetic coding. It is not a full compression tool, because it ignores all the optional lines and the MSAB header information (source name, source size, etc.). Even so, the method attained about 7% better results for the *multiz28way* data set when compared to method [24].

## Materials and methods

### 2.1 MAFCO (MAF COmpressor)

Recently, we proposed a compression method based on a mixture of finite-context models and arithmetic coding, for compressing the MSABs [11, 28]. This compressor algorithm was designed to compress only the DNA bases and alignment gaps of the ‘s’ lines, without considering other information, nor the possible presence of lower case DNA bases. On average, this algorithm was approximately 7% better than the method described in [24]. In terms of encoding time, both methods were very similar.

MAFCO is a full lossless compressor, capable of processing all line types that are part of MAF. Instead of using a mixture of finite-context models, we opted by a single model, in order to improve the encoding/decoding time. Our compression tool relies on probabilistic models, known as finite-context models, that are quite effective for DNA data compression [5]. The goal of these probabilistic models is to assign probability estimates for the next symbol, taking into account a recent past context. The size *k* of that context can vary (*k* > 0) and it will define the model order. Assuming that the *k* past outcomes are given by *x*_*n*−*k*+1..*n*_ = *x*_*n*−*k*+1_…*x*_*n*_ (order-*k* model), the probability estimates *P*(*x*_*n*+1_∣*x*_*n*−*k*+1..*n*_) are computed using the symbol counts that are accumulated while the information source is processed, with

$$P\left(s|x_{n-k+1..n}\right)=\frac{c(s|x_{n-k+1..n})+\alpha }{c(x_{n-k+1..n})+|𝓐|\alpha },$$ (2)

where *c*(*s*∣*x*_*n*−*k*+1..*n*_) represents the number of times that, in the past, symbol *s* was found having *x*_*n*−*k*+1..*n*_ as the conditioning context, ∣𝓐∣ denotes the size of the coding alphabet 𝓐, and where

$$c\left(x_{n-k+1..n}\right)=\sum_{a\in 𝓐}c\left(a|x_{n-k+1..n}\right)$$ (3)

is the total number of events that has occurred so far in association with context *x*_*n*−*k*+1..*n*_. Parameter *α* allows balancing between the maximum likelihood estimator and a uniform distribution (when the total number of events, *n*, is large, it behaves as a maximum likelihood estimator). For *α* = 1, (2) is the well-known Laplace estimator.

MAFCO uses several different types of single finite-context models. They are different in terms of order (size of the context) and in terms of alphabets that they handle. In order to find the optimal size of each finite-context model, we performed some experiments using three representative MAF files of each data set. These optimal sizes are used by default. In the following sections, we address the compression of each one of the four line types in more detail.

#### 2.1.1 Compression of the ‘s’ lines

**Fig 3:**
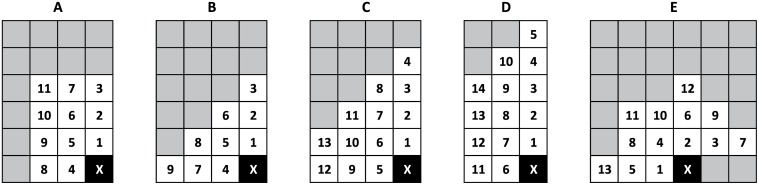
Set of 2D context templates available in MAFCO. The ‘X’ denotes the current symbol that is being encoded/decoded. Template ‘C’ with depth 10 is the default.

Similar to our previous compressor [11], MAFCO treats each MSAB as a special image type with five intensities (alphabet 𝓐_*s*_ = {A, C, G, T, -}) that correspond to the four DNA bases and the alignment gap (‘-’). MAFCO provides a set of context templates that can be used for compressing the 𝓐_*s*_ symbols of the ‘s’ lines. To improve speed, the proposed tool uses only a single model to encode the alignments of the ‘s’ lines, so only one of the context templates is used (see Fig. 3). The ‘s’ lines of each MSAB can also have other symbols than the ones indicated earlier in alphabet 𝓐_*s*_. Those other symbols include lower case symbols {a, c, g, t} and non-ACGT symbols {N, n}. So, in the ‘s’ lines the set of symbols that can be found is {A, a, C, c, G, g, T, t, N, n, -} which are mapped according to Table 1.

**Table 1:** Symbols mapping for each one of the streams illustrated in Fig. 4.

**Symbol**	**Main stream**	**Extra stream**	**Case stream**
A	0	{}	0
a	0	{}	1
C	1	0	0
c	1	0	1
G	2	{}	0
g	2	{}	1
T	3	{}	0
t	3	{}	1
N	1	1	0
n	1	1	1
-	4	{}	{}

The {} represents a stream that is not present for a particular symbol.

According to Section 1.1.1, the ‘s’ lines contain some header information that needs to be handled. The first time a given *source name* and *source size* field appears, the encoder needs to encode the entire string of *source name* and the number that corresponds to the *source size*. For the other times that this same *source name* needs to be coded, the encoder only needs to send a number that will identify this *source name*. During the decoding process, this number will be used to obtain the *source name* and *source size* fields that will be stored in an auxiliary table, with the already decoded *source names* and *source sizes*. These two fields are both encoded using a finite-context model (FCM) with an uniform distribution. The *size* field can be obtained by the number of non-gaps that are in the sequence alignment of the MSAB. The *start* field is also encoded using FCM with a uniform distribution. However, this field can be also represented by an offset that can be computed as

$$\mathrm{startOffset}={\mathrm{start}}_{x}-{\mathrm{start}}_{x-1}-{\mathrm{size}}_{x-1},$$ (4)

where start_*x*_ represents the current start of a given source, start_*x*−1_ indicates the previous start of the same source and size_*x*−1_ represents the size of the align sequence of the previous ‘s’ line of the same source. Instead of encoding the absolute *start* value, MAFCO encodes the offset using (4). This technique allows the encoder to spend less bits encoding this field, due to the fact that usually this offset is zero most of the time. This approach is only used if a ‘s’ line of a given source was already processed (a reference *start* value is necessary to compute the offset). There are also situations where the obtained offset is negative. This situation is caused because the alignment made of a given source can start at a position after the *start* position of the last alignment. In this case, MAFCO also encodes the *start* field as an absolute value. An auxiliary binary stream is needed to differentiate an absolute start value from a offset start value. This auxiliary stream is encoded using a 5-order FCM. The model order of the previous stream and all the other models that are used in MAFCO can be specified by the user, however default values are defined. The *strand* field can only have two different values, ‘+’ or ‘-’. This field is encoded using a 3-order FCM.

**Fig 4:**
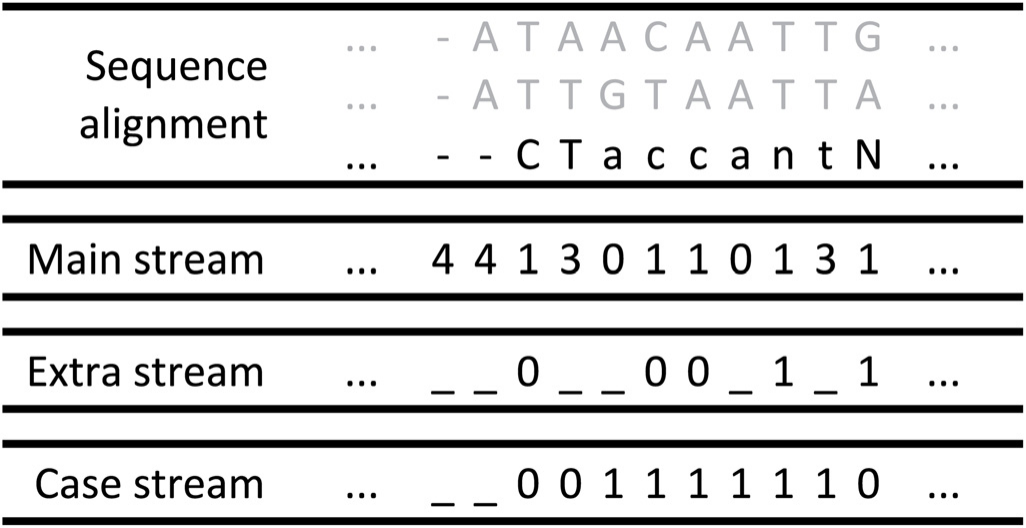
Illustration of each one of the streams used for encoding the DNA alignments information. In this example we are processing a MSAB with three lines and currently the third line is the one being processed. Depending of the symbol, the sequence can be split into at most three streams. The first stream corresponds to the DNA bases and gaps. The second stream represents the extra symbols (N’s and n’s). The last stream is used to process the upper/lower case information. All these streams are encoded using FCM and arithmetic coding.

In Table 1 we can find the symbols mapping for the DNA bases and alignment gaps along the three streams used to encode the alignment sequence. The {} represents a stream that is not present to encode a given symbol. According to Fig. 4, MAFCO splits the DNA alignments into two or three different streams. The *main stream* (always present) is a 5-symbol information source 𝓐_*s*_, which conveys the information of the DNA bases and alignment gaps. This stream is encoded using one of the five templates depicted in Fig. 3. By default, MAFCO uses the template ‘C’ with depth 10 (model order). The second stream depicted in Fig. 4 as *extra stream*, is present in absence of ACGT symbols (N’s and n’s). In case of having an alignment gap (‘-’), this *extra stream* is not necessary. This particular stream must be present to disambiguate the occurrence of the “1” symbol in the *main stream*. A “0” in this stream represents a c/C base, whereas a “1” means an extra symbol n/N. This stream is encoded by default using a 5-order FCM. As mentioned before, the MSABs may contain upper and lower case DNA bases. In order to encode this information, a third stream, called *case stream*, is necessary. This binary stream is associated to each symbol of the *main stream* (except the alignment gap symbol ‘-’), indicating the respective case type. Similar to the previous streams, this stream is also compressed using a order-5 FCM.

#### 2.1.2 Compression of the ‘q’ lines

As mentioned in Section 1.1.1, the ‘q’ lines contain information about the quality of each aligned base of the immediately preceding ‘s’ line. The source name is the same as the previous ‘s’ line, so that information was already processed before and it is not necessary to encode it again. Regarding the quality values, in this case we have an alphabet of 13 symbols: 𝓐_*q*_ = {0, 1, 2, 3, 4, 5, 6, 7, 8, 9, F, −, .}. In fact, the size of the alphabet is 12 symbols, because the gap positions are the same as in the immediately preceding ‘s’ line, so the alignment gaps are not encoded here. This stream of quality values is encoded using a single order-5 FCM. A different model order can be specified by the user, but for a large alphabet such as 𝓐_*q*_, higher model orders require more memory. Furthermore, the presence of these ‘q’ lines needs to be encoded as well, because there are MAF files that may not have these optional lines. A binary stream is then necessary to indicate, for each MSAB, if it contains ‘q’ lines. Inside each MSAB, it is also necessary to indicate for each ‘s’ line if there is a ‘q’ line associated (having the same source name). The first ‘s’ line (reference source) does not have any ‘q’ line associated. However, for the remaining ‘s’ lines, it is necessary a second binary stream that indicates if it has a ‘q’ line associated. These two binary streams are encoded also using a 5-order FCM.

#### 2.1.3 Compression of the ‘i’ lines

Similar to the ‘q’ lines, the ‘i’ lines are also associated to a ‘s’ line. This means that the source name of a ‘i’ line is the same as the immediately preceding ‘s’ line. The only information that needs to be encoded corresponds to the four fields described in Section 1.1.1. The counts are compressed using a single FCM with a uniform distribution. The status symbols are encoded using a 4-order FCM.

After analyzing the contents of some of the MAF files, we noticed that there is a correlation between the left and right status and counts of the same source. Basically, the left status symbol and count of a given source is the same as the last right status symbol and count. This means that we only need to encode both left and right status and count for the first time that a given source of a ‘i’ line appears. After the first occurrence of a ‘i’ line of a given source, the proposed method only encodes the right status and count. The left status and count can be obtained by the previous right status and count already processed. However, there are some irregularities in this “rule”. Sometimes, the left status or/and count are different from the previous right status and count. In order to overcome these irregularities, we created two auxiliary binary streams that represent these irregularities (one stream for the irregular counts and the other for the irregular status symbols). Both streams are independent, because it is possible to have only an irregular count and not an irregular status symbol. By default, these two streams are encoded using a 5-order FCM. Similar to the ‘q’ lines the ‘i’ lines also have a binary stream that indicates if the current MSAB has ‘i’ lines. This stream is also encoded using a 5-order FCM.

#### 2.1.4 Compression of the ‘e’ lines

The ‘e’ lines are quite different, when compared to the ‘q’ and ‘i’ lines. They are not associated with any of the ‘s’ lines of the MSAB that is being encoded. However, they only appear in a MSAB if in any of the previous MSABs a ‘s’ or a ‘e’ line of the same source occurred. Because the ‘e’ lines are not associated to any ‘s’ lines of the current MSAB, all header fields (source name, start position, etc.) need to be encoded. This header information is compressed in the same way as the header information of the ‘s’ lines. The ‘e’ lines have a status field that the ‘s’ lines do not have. The status field of a given ‘e’ line usually has the same value of the status field as the ‘e’ line of the immediately preceding MSAB or the last ‘i’ line processed of the same source. Two auxiliary binary streams are necessary to encode the status symbol of a given ‘e’ line. These streams indicate if an irregularity occurred between the current status value of a given ‘e’ line and the previous ‘e’ or ‘i’ line status value of the same source. Both streams are encoded using a 5-order FCM.

#### 2.1.5 Parallel processing and partial decoding

Genomic data files are growing in size every day, a growth that leads to both storage and access issues. In order to overcome these issues, MAFCO uses an approach that allows large files to be split into several parts that can be compressed/decompressed in parallel. This approach reduces the compression/decompression time. However, some compression ratio loss might occur, because statistics gathered in one part of the file may not be available in other parts. Despite this, the compression ratio loss in large files is usually small, and largely compensated by the gain in compression/decompression time. Furthermore, this splitting approach allows the user to decode only some parts of the encoded file, without needing to decode the full compressed file.

The proposed compression tool allows parallel compression/decompression of MAF files that contain several MSABs. After the splitting process, each part contains an integer number of MSABs. By default, the compression tool splits the input MAF file into four parts and, by consequence, uses also 4 threads. The number of parts in which the MAF file can be split may be specified by the user (-ng flag), as well as the maximum number of threads (-nt flag). We call to each part of the split file a GOBs (Group Of Blocks). By default, in the decoder the entire compressed file is decoded. However, the user can specify a range of GOBs to decode (-ng flag). Note that it is only possible to decode a range of GOBs if the file was initially encoded using a multi-part approach. This approach is quite useful, because it can reduce the decompression time, by skipping the decoding of some unneeded GOBs. Furthermore, the number of threads that is used in the decoder does not have to be the same as in the encoder. This allows, for example, the compression of large files using multi-core computers, while being able to decompress them in more modest machines, if needed. This capability is helpful, because usually the compression of MAF files is done only once by a powerful multi-core computer. The decoding is done many times by the research community, using computers with very different capabilities.

### 2.2 Data sets

We used four data sets for assessing the performance of the proposed compression tool. The data sets were retrieved from the UCSC Genome Bioinformatics Browser (see S2 Table). The four data sets used are aligned taking as a reference the human genome, although the proposed compression tool is compatible with other data sets. For example there are data sets that are aligned with other species such as: chicken [29], mouse [30], gorilla [31], etc. We decided to only include the data sets that have the human genome as a reference because they are the most relevant ones used in the literature.

The data set *multiz28wayB* was created from the “multiz28wayAnno.tar.gz” file, which contains alignments similar (but not equal) to the ones in *multiz28way*, with the optional ‘q’, ‘i’ and ‘e’ lines that are not present in the *multiz28way* data set.

The *multiz28way* data set was first used by Hanus *et al*. [24]. It contains 27 vertebrate genomes aligned with the human genome (a total of 28 species). The *multiz46way* and *multiz100way* contain 45 and 99 vertebrate genomes, respectively, also aligned with the human genome (a total of 46 and 100 species respectively). These data sets are quite different in terms of number of species and consequently in terms of size. Moreover, they are also different in terms of the line types that each one contains.

In Section 1.1.1 we describe the line types that can be found in MAF files. In Table 2, we can see the different line types that each data set has. The ‘s’ lines are the most important lines so they appear in all the four data sets. The *multiz28wayB* and *multiz46way* data sets are the only ones that have ‘q’ lines. The ‘i’ and ‘e’ lines can only be found in the *multiz28wayB*, *multiz46way*, and *multiz100way* data sets. These differences will affect the attained compression results, as we will explain in the results section.

**Table 2:** Approximate raw size of each data set, number of species, number of MSABs, and line types that each data set has.

**Data set**	**Uncompressed size (gigabytes)**	**Number of species**	**Number of MSABs**	**Line types**			
				**‘s’**	**‘q’**	**‘i’**	**‘e’**
*multiz28way*	45	28	23, 120, 374	Yes	No	No	No
*multiz28wayB*	106	28	23, 387, 797	Yes	Yes	Yes	Yes
*multiz46way*	252	46	33, 429, 985	Yes	Yes	Yes	Yes
*multiz100way*	716	100	109, 850, 940	Yes	No	Yes	Yes

“Yes” symbolizes the presence of a line type in the data set while “No” symbolizes absence.

#### 2.2.1 Statistical information of the data sets

This section presents some statistical information regarding the data sets used in this work. The locations where these data sets were retrieved are depicted in S2 Table.

There are some important differences among the data set used in this work that are worth to describe in order to justify some of the obtained results. In what follows we will present some statistics of the data sets used in this work. One interesting and important statistic (depicted in S1 and S2 Figs.) is the size of each MSAB. From S1 Fig., we conclude that, in terms of percentage of occurrence, there is a similar statistical pattern in the four data sets. The maximum number of rows of the first two data sets is 28, because it corresponds to the number of species in these data sets. For the other two data sets, the maximum number of rows is 46 and 100, respectively. Despite the first two data sets have the same number of species and the species are also similar, the statistics regarding the number of rows in each MSAB is different. We can see in S1 Fig. that the *multiz28wayB* has more MSABs with one row, compared to the *multiz28way* (about 1.1% more). There are also small differences (lower than 0.1%) between those two data sets for higher number of rows.

Regarding the number of columns of each MSAB, in S2 Fig. we can find some statistics for the data sets used in this work. For the first three data sets (*multiz28way* *multiz28wayB*, and *multiz46way*), we notice a similar statistical pattern. However, the last data set (*multiz100way*) has a very different statistical pattern. This difference affects the compression results (in Section 3 we will address this subject again). Contrarily to the first three data sets, there is a high percentage of MSABs (about 13.8%) than only have one column in *multiz100way*. These MSABs with few columns impact the compression results of the proposed method, as we will see in Section 3. It is important to note that the range of the number of columns for each MSAB is quite large. For example, in *multiz100way* the maximum number of columns for a MSAB can go up to 11, 236, 519. In order to create the charts of S2 Fig., we filtered the statistics in order to contain about 90% of the occurrences. This way, we can analyze more easily the obtained results. Regarding the first two data sets, there are again some minor statistical differences, lower than 0.1%, that are relevant in terms of compression results.

#### 2.2.2 Statistical information of the ‘s’, ‘q’, ‘i’, and ‘e’ line types

The ‘s’ lines contain the DNA alignments. These alignments are usually DNA bases that include the nucleotides {A, C, G, T}. However, there are some other symbols in these particular data sets which may include some non-ACGT characters (N’s and the alignment gap ‘-’), as well as upper and lower case characters. S3 Fig. shows the statistics for the set of characters that occur in the ‘s’ lines. We can see that the N’s characters are the less frequent. On the other hand, the gap symbol (‘-’) is the most frequent symbol. The frequency of the gap symbol tends to increase when the number of species increases. This is justified by the increased difficulty in creating alignments with more species, leading to more alignment gaps.

Regarding the ‘q’ lines and according to what we mentioned in Section 1.1.1, there are 11 different quality values that may occur. There are also other possible characters like the alignment gap ‘-’ and a dot character ‘.’, that indicates a missing quality value. In S4 Fig., we can find the statistics of each one of the 11 quality values present in the *multiz28wayB* and *multiz46way* data sets. Notice that the other two data sets do not have ‘q’ lines. According to S4 Fig., the quality value 9 occurs more than 92%, meaning that the majority of the alignments have a high quality level (see S1 Table).

The ‘i’ lines have two of the fields called left and right status. According to what we have described in Section 1.1.1, there are six different status characters. In S5 Fig., we can find the percentage of occurrence of each status character. We did not take into account the left and right status, since we have simply computed the overall percentage (left and right together). The most frequent status character is ‘C’. This means that there is an high percentage of contiguous sequences between entire MSABs, i.e., it exists some redundancy between several MSABs.

Finally in the ‘e’ lines, there is also a status character field, but there are only five possible characters in this case (S6 Fig. depicts their percentage of occurrence) As we can see, the most frequent status character is ‘I’, which symbolizes the presence of non-aligning bases in the source species between chained MSABs before and after this MSAB.

## Results and discussion

The experimental results described in this section were performed in a Linux server running Ubuntu with 16 Intel(R) Xeon(R) CPUs (E7320 2.13GHz) and 256 gigabytes of memory. The source code and two Windows binaries (32 and 64 bits) are available for testing at http://bioinformatics.ua.pt/software/mafco. There are also three small MAF files available at this site, as well as instructions of how to quickly test the compression program.

In this section, we present the compression results obtained using several popular general compression methods, such as gzip, bzip2, ppmd, and lzma (the last two using the version implemented by the 7z archiver) as well as by method [24], the maf-bgzip tool [25–27] and by the compression tool described in this paper.

The performance of each compression method can be found summarized in Table 3. Alternative, in S3–S6 Tables we can find the same results with more detail for each MAF file for the four data sets used in this work. The results are presented in bytes for gzip and in percentage compression gain in relation with gzip, for the other methods. The compression gain in relation with gzip was computed as:

$$G_{M}=100\times \frac{{\mathrm{NBytes}}_{gzip}-{\mathrm{NBytes}}_{M}}{{\mathrm{NBytes}}_{gzip}}$$ (5)

where *M* denotes a compression method and the “NBytes” corresponds to the size of the compressed file in bytes. These results were obtained using a single thread and without splitting the MAF file in several GOBs, and will be used later to evaluate the performance loss when the compressor splits the input MAF file into several GOBs. For the *multiz28way*, the proposed method attained about 9% better results, when compared to Hanus *et al.* method [24]. It was not possible to obtain the compression results for the other data sets, using method [24], due to compatibility problems. When compared with gzip, MAFCO attained a compression gain of 51.7% for the *multiz28way* data set. For the *multiz28wayB* and *multiz46way* data sets, the compression gain is about 54.3% and 57.3%, respectively. Without considering the *multiz100way* data set, it seems that the compression gain tends to increase with the size of each data set. The lower compression performance (about 34.1%) attained in the *multiz100way* data set using MAFCO can be justified due to the small average number of columns of the MSABs (see S3 Fig.), suggesting that the model presented in Section 2.1.1 to compress the ‘s’ lines is less effective when the MSABs have a small number of columns.

**Table 3:** Performance of several compression methods in the four data sets used in this work.

Data set	Original Size	GZIP size	BZIP2	PPMD	LZMA	BGZIP	MSAc	MAFCO
*multiz28way*	48, 510, 921, 185	10, 443, 713, 974	8.7%	10.9%	23.1%	-21.1%	46.8%	51.7%
*multiz28wayB*	113, 528, 207, 035	16, 216, 614, 098	16.6%	13.7%	20.7%	-35.1%	–	54.3%
*multiz46way*	270, 579, 509, 536	32, 523, 764, 993	18.1%	5.1%	21.0%	-49.1%	–	57.3%
*multiz100way*	794, 243, 994, 061	72, 086, 319, 647	21.2%	-8.5%	20.7%	-79.7%	–	34.1%
Total	1, 226, 862, 631, 817	131, 270, 412, 712	18.9%	-0.8%	21.0%	-61.9%	–	43.7%

“BGZIP” refers to the maf-bgzip tool [25–27], “MSAc” refers to method [24] and MAFCO is the proposed method. Size is presented in bytes, whereas the percentages indicate the amount of reduction attained in comparison to gzip.

Looking again at Table 3, we can see that the performance of bzip2 increases as the size of the data sets increases. The ppmd and maf-bgzip performance have a different behavior: it decreases as the data sets increase in size, even reaching “negative” performances for the *multiz100way* in case of the ppmd. The maf-bgzip has the worst performance for all data sets, when compared to gzip. The reason to this low performance is due to the nature of the compression method. As mentioned earlier in Section 1.2, the goal of this tool is to provide fast random access to gzip files, sacrificing compression performance. Method [24] works only for data sets having exclusively ‘s’ lines (e.g., the *multiz28way* data set). Despite the good results when compared to gzip (about 46.8%), the compression tool provided by Hanus *et al.* does not work in some files of the *multiz28way* data set (*chr15*, *chr16*, *chr17*, and *chr19*). The encoder is capable of encoding those files, however it was not possible to decompress them.

**Fig 5:**
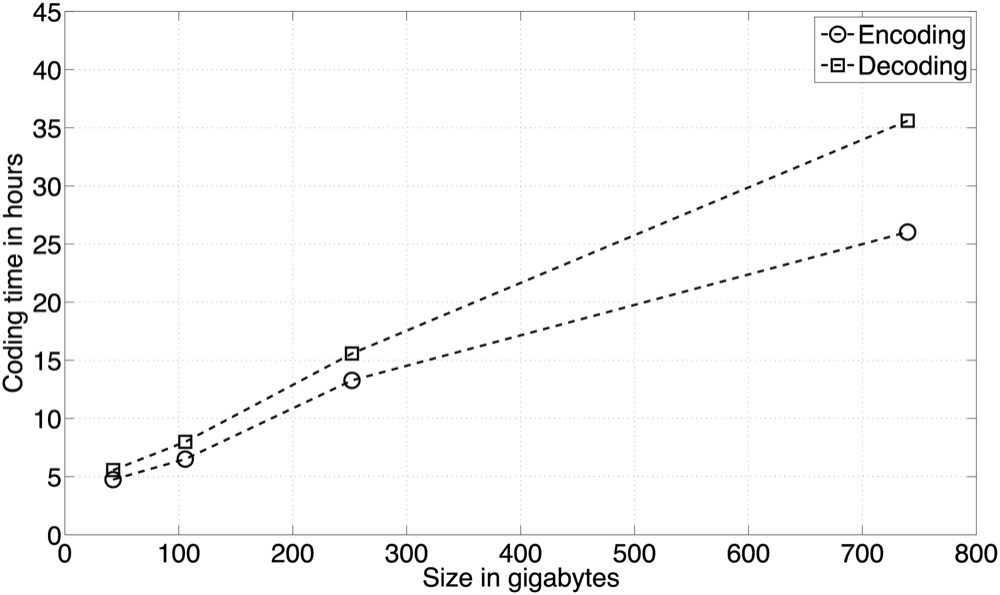
Relation between the size of each data set and the encoding/decoding time in hours. Each marker represents a data set (from left to right the markers correspond to the *multiz28way*, *multiz28wayB*, *multiz46way*, and *multiz100way* data sets). There is almost a linear behavior through the four data sets. The slightly deviation from the linear behavior is due to the difference between each data set in terms of line types.

In terms of global coding time (compression plus decompression), our method is slower when compared to all the other methods, except method [24]. However, it seems that in the encoding phase lzma is the slowest method among all the others. Despite all this, the proposed compression tool is ≈ 5 times faster than method [24]. In the decoding phase, MAFCO is ≈ 4 times faster, when compared to the Hanus *et al.* method [24]. These conclusions were only made based on the results of S3 Table for the *multiz28way* data set, using a single thread and without splitting the MAF file in several GOBs. Fig. 5 illustrates the relation between the size of each data set and the corresponding encoding/decoding time using a single thread.

The obtained curves are almost linear through the four data sets. We can notice a slight deviation due to the differences between each data set in terms of line types that will affect the obtained results. Moreover, we can also observe that the time difference between encoding and decoding tends to increase for larger data sets.

As mentioned earlier in Section 2.1.5, MAFCO implementation allows the user to split the MAF file into several GOBs and also to encode/decode them in parallel, using several threads. Table 4 presents the performance of MAFCO compared to gzip, when we split the MAF file into 1, 2, 4, and 8 GOBs. It is easy to conclude that even when the MAF file is split into 8 GOBs, the compression loss is lower than 1%, when compared to the results where the MAF file was not split.

**Table 4:** Compression performance of MAFCO using 1, 2, 4 and 8 threads/GOBs for the four data sets used in this work.

**Data sets**	**Number of threads/GOBs**			
	1	2	4	8
*multiz28way*	51.74	51.51	51.20	50.78
*multiz28wayB*	54.34	54.17	53.94	53.62
*multiz46way*	57.29	57.18	57.02	56.81
*multiz100way*	34.05	34.00	33.93	33.84
Overall	43.72	43.63	43.50	43.32

The performance in percentage is in relation to gzip and was computed according to Equation (5) illustrated in page 10.

The results of Table 5 correspond to the total amount of time needed to encode/decode the four data set using 1, 2, 4 and 8 threads. For individual results for each data set, consult S7–S10 Tables. The obtained results were obtained by splitting the input MAF file into 1, 2, 4, and 8 GOBs. We used the same number of threads as the number of GOBs in our simulations, although MAFCO is capable of encoding/decoding a MAF file with a number of parallel processes that is different from the number of parts in which the file was split. Furthermore, the decoder can also use a number of threads that is different from the number used during encoding. In this particular case, we used the same number of threads, in order to be easier to analyze the results obtained. In term of encoding/decoding time we present three measures: “CPU time”, which corresponds to the total system and user time obtained by the Linux *time* function; The “Optimal CPU time”, computed by dividing “CPU time” by the number of threads used. The left values correspond to the encoding stage, whereas the right ones correspond to the decoding process. The previous time measures were used to compute the *speedup* and *efficiency* metrics. The first metric is defined as

$$S_{p}=\frac{T_{1}}{T_{p}},$$ (6)

where *p* corresponds to the number of parallel processes, *T*_1_ is the execution time using one thread (sequential algorithm) and *T*_*p*_ is the execution time using *p* parallel processes. Linear or ideal speedup is obtained if *S*_*p*_ = *p*. The efficiency metric can be computed as

$$E_{p}=\frac{S_{p}}{p}=\frac{T_{1}}{pT_{p}}.$$ (7)

This metric is a value between zero and one, that indicates how well-used the processors are in executing the algorithm. Efficiency values close to one correspond to linear speedup algorithms. On the other hand, values close to zero indicate that the processors are not being well-used (poor parallelization).

**Table 5:** Performance of MAFCO using 1, 2, 4 and 8 threads for the four data sets used in this work.

**Measure**	**Encoding**				**Decoding**			
Number of threads	1	2	4	8	1	2	4	8
CPU time (secs)	171, 325	172, 534	175, 087	184, 358	236, 992	239, 751	241, 855	243, 996
Optimal CPU time (secs)	171, 325	86, 267	43, 772	23, 045	236, 992	119, 876	60, 464	30, 500
Speedup	1.00	1.99	3.91	7.43	1.00	1.98	3.92	7.77
Efficiency	1.00	0.99	0.98	0.93	1.00	0.99	0.98	0.97

The CPU time corresponds to the total CPU time obtained by the *time* command in Linux. The optimal CPU time corresponds to the CPU time divided by the number of threads. The speedup was computed by dividing the optimal CPU time for one thread (sequential execution) by the optimal CPU time for *n* threads (calculated according to (6)). Finally, the efficiency is obtained by dividing the speedup by the number of threads (according to (7)).

Analyzing the obtained results, we can see that MAFCO has a linear speedup, up to 8 parallel processes. It seems that the efficiency of our method is similar between the encoding and decoding phases, regardless of the number of parallel processes used (up to 8 in this case).

**Fig 6:**
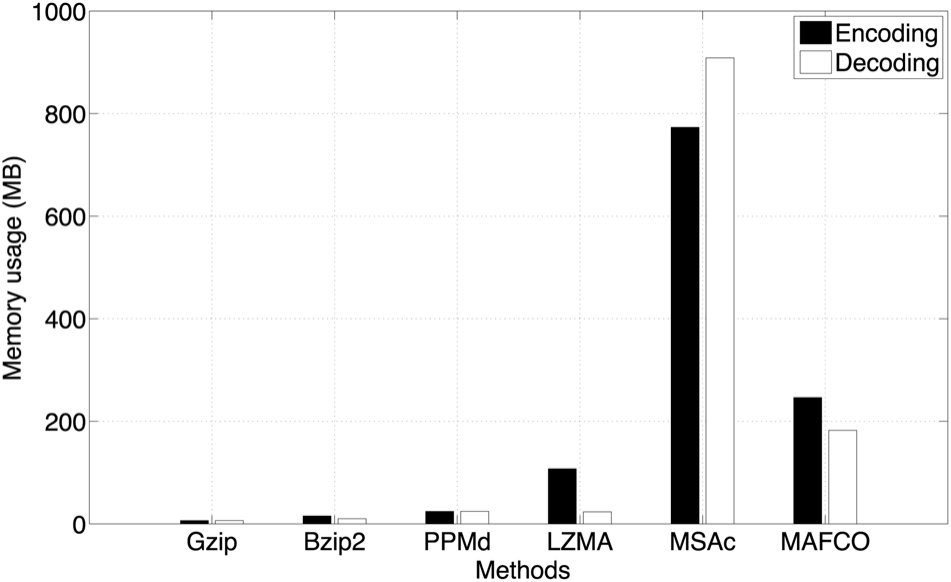
Encoding and decoding memory usage in megabytes for all methods used in this work (except maf-bgzip). The memory has been estimated with *valgrind*, using *massif*. The presented values were computed considering only three representative MAF files (*chr2*, *chr9*, and *chrY*) from the *multiz28way* data set.

Fig. 6 depicts the encoding/decoding memory usage in megabytes for all the methods (except maf-bgzip). The memory has been estimated with *valgrind*, using *massif*. Because *valgrind* is very slow, we decided to assess the memory usage using three files from the *multiz28way* data set (*chr2*, *chr9*, and *chrY*). We computed the average value between the three files and plotted the obtained results in Fig. 6. As can be seen, along the methods evaluated, gzip, bzip2 and ppmd are the ones that require less memory. Method [24], denoted in Fig. 6 as “MSAc”, is the one that requires more memory. The proposed method requires less memory than method [24] but more than lzma.

## Conclusions

It is clear that gzip is still wide used by the research community to compress genomic data due to is simplicity, pervasiveness and relatively good speed. Nevertheless, we cannot ignore the fact that the performance of gzip is limited in terms of compression gain because it is a general compression algorithm that was not created to compress specific genomic data. In this manuscript we present a compression method capable of attain file reduction close to 50% or even more, in relation to gzip. This means that it is virtually possible to store twice as much data in the same storage space available, by using a different compression tool. The utilization of more sophisticated compression algorithms is then worthy of consideration by the genomic laboratories. Higher compression results usually require complex algorithms that usually need more time and memory to run. However, these additional requirements are almost always compensated by the gains in terms of available storage. In summary, we are confident that the compression tool presented in this article is a relevant contribution for slowing down in part the data deluge that we are facing nowadays.

## Supporting Information

S1 Fig Number of rows of each MSAB for the *multiz28way*, *multiz28wayB*, *multiz46way*, and *multiz100way* respectively. The results are depicted in terms of percentage of occurrence in order to be more easy to analyze them. Taking into account the maximum number of rows per MSAB allowed for each data set is 28, 28, 46, and 100 respectively, there is a similar statistic pattern between the four data sets.
(EPS)

S2 Fig Number of columns of each MSAB for the *multiz28way*, *multiz28wayB*, *multiz46way*, and *multiz100way* respectively. The results are depicted in term of percentage of occurrence and only cover about 90% of the occurrences in order to be more easy to make some conclusions. The first three data sets have a similar statistical pattern. On the contrary, in the last data set the statistical pattern is quite different when compared to the first two data sets.
(EPS)

S3 Fig Statistic of the set of characters that occur in the ‘s’ lines for the four data sets used in this work. Results are presented in percentage.
(EPS)

S4 Fig Percentage of occurrence of each one of the 11 possible quality values in the ‘q’ lines for the *multiz28wayB* and *multiz46way* data sets. The *multiz28way* and *multiz100way* do not have ‘q’ lines.
(EPS)

S5 Fig Percentage of occurrence for each of the six possible status symbols in the ‘i’ lines for the *multiz28wayB*, *multiz46way*, and *multiz100way* data sets. The *multiz28way* does not have ‘i’ lines.
(EPS)

S6 Fig Percentage of occurrence for each of the five possible status symbols in the ‘e’ lines for the *multiz28wayB*, *multiz46way*, and *multiz100way* data sets. The *multiz28way* does not have ‘e’ lines.
(EPS)

S1 Table Mapping of MAF quality values. The quality values can be ‘F’ (finished sequence) or a number derived from the actual quality scores (which ranges from 0–97) or the manually assigned score of 98. The MAF quality values are computed according to (1).
(EPS)

S2 Table Data sets information used in this work. The four data sets are aligned using the human specie as a reference. The data set were retrieved from the UCSC database. The *multiz28wayB* was created from the “multiz28wayAnno.tar.gz” file which contains almost (the alignments are slight different in some MSABs) the same alignments of the *multiz28way* for the same species for all chromosomes, with additional annotations to indicate gap context, genomic breaks, and quality scores for the sequence in the underlying genome assemblies (optional ‘q’, ‘i’ and ‘e’ lines).
(EPS)

S3 Table Performance of several compression methods in the *multiz28way* data set. “BGZIP” refers to the maf-bgzip tool [25–27], “MSAc” refers to the [24] method and MAFCO is the proposed method. Size is indicated in bytes, whereas the percentages indicate the amount of reduction attained in comparison to gzip. “CTime” and “DTime” indicate the compression and decompression times in seconds, respectively. It was not possible to obtain the decompression time for the “MSAc” method for files *chr15*, *chr16*, *chr17*, and *chr19* due to the inability to decompress the previous compressed files. The “MSAc” decoder crashed for those files.
(EPS)

S4 Table Performance of several compression methods in the *multiz28wayB* data set. “BGZIP” refers to the maf-bgzip tool [25–27], and MAFCO is the proposed method. Size is indicated in bytes, whereas the percentages indicate the amount of reduction attained in comparison to gzip. “CTime” and “DTime” indicate the compression and decompression times in seconds, respectively. Results for method [24] were not included because it is not able to process the files of this data set.
(EPS)

S5 Table Performance of several compression methods in the *multiz46way* data set. “BGZIP” refers to the maf-bgzip tool [25–27], and MAFCO is the proposed method. Size is indicated in bytes, whereas the percentages indicate the amount of reduction attained in comparison to gzip. “CTime” and “DTime” indicate the compression and decompression times in seconds, respectively. Results for method [24] were not included because it is not able to process the files of this data set.
(EPS)

S6 Table Performance of several compression methods in the *multiz100way* data set. “BGZIP” refers to the maf-bgzip tool [25–27], and MAFCO is the proposed method. Size is indicated in bytes, whereas the percentages indicate the amount of reduction attained in comparison to gzip. “CTime” and “DTime” indicate the compression and decompression times in seconds, respectively. Results for method [24] were not included because it is not able to process the files of this data set.
(EPS)

S7 Table Performance in terms of coding time of MAFCO using 1, 2, 4 and 8 threads for the *multiz28way* data set. The CPU time corresponds to the total CPU time obtained by the *time* command in Linux. The optimal CPU time corresponds to the CPU time divided by the number of threads. The speedup was computed by dividing the optimal CPU time for one thread (sequential execution) by the optimal CPU time for *n* threads (calculated according to (6)). Finally, the efficiency is obtained by dividing the speedup by the number of threads (according to (7)).
(EPS)

S8 Table Performance in terms of coding time of MAFCO using 1, 2, 4 and 8 threads for the *multiz28wayB* data set. The CPU time corresponds to the total CPU time obtained by the *time* command in Linux. The optimal CPU time corresponds to the CPU time divided by the number of threads. The speedup was computed by dividing the optimal CPU time for one thread (sequential execution) by the optimal CPU time for *n* threads (calculated according to (6)). Finally, the efficiency is obtained by dividing the speedup by the number of threads (according to (7)).
(EPS)

S9 Table Performance in terms of coding time of MAFCO using 1, 2, 4 and 8 threads for the *multiz46way* data set. The CPU time corresponds to the total CPU time obtained by the *time* command in Linux. The optimal CPU time corresponds to the CPU time divided by the number of threads. The speedup was computed by dividing the optimal CPU time for one thread (sequential execution) by the optimal CPU time for *n* threads (calculated according to (6)). Finally, the efficiency is obtained by dividing the speedup by the number of threads (according to (7)).
(EPS)

S10 Table Performance in terms of coding time of MAFCO using 1, 2, 4 and 8 threads for the *multiz100way* data set. The CPU time corresponds to the total CPU time obtained by the *time* command in Linux. The optimal CPU time corresponds to the CPU time divided by the number of threads. The speedup was computed by dividing the optimal CPU time for one thread (sequential execution) by the optimal CPU time for *n* threads (calculated according to (6)). Finally, the efficiency is obtained by dividing the speedup by the number of threads (according to (7)).
(EPS)
